# Comparison of combined treatment with desmopressin plus oxybutynin and desmopressin plus tolterodine in treatment of children with primary nocturnal enuresis

**DOI:** 10.12861/jrip.2015.16

**Published:** 2015-09-01

**Authors:** Anoush Azarfar, Mohammad Esmaeili, Mitra Naseri, Fatemeh Ghane, Yalda Ravanshad, Marjan Vejdani, Neda Ghanei, Akbar Babaei-Heydarabadi, Seyed-Ehsan Saffari

**Affiliations:** ^1^Department of Pediatrics, School of Medicine, Mashhad University of Medical Sciences, Mashhad, Iran; ^2^Clinical Research Development Center, Ghaem Hospital, School of Medicine, Mashhad University of Medical Sciences, Mashhad, Iran; ^3^Iranian Research Center on Healthy Aging, Sabzevar University of Medical Sciences, Sabzevar, Iran; ^4^Student Research Committee, Mashhad University of Medical Sciences, Mashhad, Iran; ^5^Department of Public Health, Ahvaz Jundishapur University of Medical Sciences, Ahvaz, Iran; ^6^Sabzevar University of Medical Sciences, Sabzevar, Iran

**Keywords:** Desmopressin, Oxybutynin, Tolterodine, Primary nocturnal enuresis

## Abstract

**Introduction:** Nocturnal enuresis (enuresis) is one of the most common developmental problems of childhood, which has often a familial basis, causes mental and psychological damage to the child and disrupts family solace. ****

**Objectives:** In this study, we compared therapeutic effects of combination therapy of desmopressin plus oxybutynin with desmopressin plus tolterodine, in the treatment of children with primary nocturnal enuresis.

**Patients and Methods:** The present study is a clinical trial study, where 59 patients with primary nocturnal enuresis in the age range of 5 to 14 years old were selected from the visitors of nephrology clinic of Dr. Sheikh pediatrics hospital (Mashhad, Iran). Patients were divided into 2 treatment groups where the first group received combined therapy with desmopressin and oxybutynin, and the second group received combined therapy with desmopressin and tolterodine. Data was analyzed using SPSS 16 software and descriptive and analytical statistics (chi-square test).

**Results:** The mean of age of patients in total was 2.55 ± 7.90 years. In the treatment group with desmopressin and oxybutynin, 26 of 30 patients (86.7%) achieved a complete remission and 4 patients (13.3%) still suffered from enuresis during a 3-month evaluation. The comparison of 2 groups, in terms of the outcome of the 3-month treatment, showed significant differences between the remission and recovery of 2 groups, where the recovery in the group with desmopressin plus tolterodine was higher than the group with desmopressin plus oxybutynin (*P* = 0.001).

**Conclusion:** The results showed that combined treatment with desmopressin plus tolterodine performs better than desmopressin plus oxybutynin .

**Implication for health policy/practice/research/medical education:**

Nocturnal enuresis is known as one of the most common problems in children with several negative impacts on the child and his/her family. In a study, we found the significant treatment improvement of nocturnal enuresis by prescribing oxybutynin and tolterodine, therefore, it is recommended that health professionals take this combined treatment into account for a higher efficiency.

## Introduction

Nocturnal enuresis (enuresis) is one of the most common developmental problems of childhood which has often a familial basis. It is usually benign and gradually disappears with age (1). Five-year old children should be able to control their urine (1) because at this age, the bladder capacity increases, and the nerve centers of the brain are able to control bladder contractions (2,3). The involuntary excretion of urine, at least 2 times a week for 3 consecutive months and the chronological age of five years (4) without any apparent congenital or acquired defect, is called nocturnal enuresis that may be classified as primary or secondary (1).

Enuresis is considered primary when the child never had dry bedding for six months (5), and it is considered secondary when, at least for 6 months, the child had dry bedding. Most cases of enuresis (85%) are of the primary type (1). So far, no clear causes of enuresis have been determined (5), but in terms of etiology, various factors may be involved in its occurrence including sleep disorders, emotional stress, hereditary background, small functional capacity of bladder, and non-inhibitory contractions of detrusor.

Among the organic causes, we can refer to urinary infection, reflux or urinary tract anomalies noted (6,7). In general, the cause of most cases of primary enuresis is the delay in the development and function of the bladder (8). Urine control is rare before the age of 18 months old. After that, until 4.5 years old, the child each year acquires 20% of ability to control urine (9). The enuresis in children of 5 years old is therefore 15% to 20% (10), in children of 8 years, it is 4% and in children of 14 years old, it is 1% (11). Sixty-five percent of children with enuresis are male, and the family history is positive in 50% of cases. In children with enuresis, it is observed that 15% of spontaneous recovery is achieved each year (12). In the United States, approximately 5 million children are affected by this problem, and in the studies conducted in the United States, 61% of parents have reported that their biggest problem is the enuresis of their children, which have caused children to be punished (13).

Enuresis treatment includes medicinal and nonmedicinal treatments. Nonmedical treatment includes motivational therapy, behavioral therapy and training and strengthening the bladder. Medicinal treatment includes imipramine, anticholinergics, desmopressin and oxybutynin, singly or combined together (14,15). Considering the fact that enuresis has a prevalence of 20% and causes a lot of anxiety in the child and his/her family, and that in 25% of cases enuresis is resistant to treatments with desmopressin, new therapy methods are indispensable (16). Therefore, in the present study we investigate the effect of combined treatment with desmopressin and oxybutynin versus the treatment with desmopressin and tolterodine, conducted in Dr. Sheikh Hospital (Mashhad, Iran).

## Patients and Methods

The present study is a clinical trial study conducted on patients with primary nocturnal enuresis in age range of 5 to 15 years who were selected from the visitors of the nephrology clinic of Dr. Sheikh pediatrics hospital in 2012. Sixty children with enuresis, aged 5 to 14 years, were randomly selected. Exclusion criteria included patients with neurogenic bladder (such as tumoral lesions, spinal cord injury, or sacrococcygeal congenital abnormalities and urogenital anomalies), CNS disorders (such as cerebral palsy [CP] and mental retardation [MR]), cases of secondary enuresis, urinary concentrating defects (including renal tubules dysfunction, electrolyte imbalances, diabetes [DI–DM]) and reluctance of the patient or his/her parents to participate in the study or to continue. After performing diagnostic tests by the nephrology specialist for screening of primary nocturnal enuresis from other cases, the cases were randomly divided into 2 groups. The study approach was as follows:

(*A*) Complete description of 1) associated symptoms (primary or secondary) (night or day) (urinary symptoms), 2) evolutionary history: the time of acquisition of developmental and controlling measures of bladder and 3) family history: history in the parents, sister, brother and horrible events.

(*B*) Physical examination: 1) examination of external genitalia, 2) examination of abdomen for kidney or bladder mass and 3) neurological examination: peripheral reflexes, sensation, sense of the perineal region.

(*C*) Experimental study: 1) Urinalysis for the measurement of specific gravity, glucose, blood, protein 2) urine culture, 3) blood glucose, 4) serum creatinine, 5) renal ultrasonography.

After gaining confidence about the diagnosis of primary nocturnal enuresis, the patients were placed into 2 groups. For the first group (n = 30 patients), the combined treatment of desmopressin and oxybutynin and for the second group (n = 30 patients), the combined treatment of desmopressin and tolterodine was prescribed. Since oral desmopressin tablets were more costly and not affordable for all patients, for homogenization of cases under study, desmopressin nasal spray was prescribed, for all the patients. In addition, oral oxybutynin tablets were prescribed. Patients’ contact information was recorded, and the parents were advised to continue medication, as prescribed, for 3 months. They were also advised to inform the medical team in case of any problem such as drug incompatibilities. The demographic data was confidential and is not used in reports. It is worth noting that both methods used in this study were previously studied in terms of complications and application in the treatment of enuresis, and the obtained results indicate their efficacy for the treatment of enuresis in children. In the monthly visits to the nephrology clinic, the patients were monitored by nephrologists in charged in the project, in order to be examined for the drug side effects and enuresis recovery or reduce of the urination frequency during the night. Drug side effects were evaluated in the form of presence or absence of any complications in the patients who during the study, due to the intake of the drug visited the clinic with the concerned clinical complaints that had already been explained to them.

The final outcome was improvement in nocturnal enuresis which included less than twice a month enuresis in children who had had this problem. It should be noted that due to the lack of facilities and practical limitations there was no possibility to operate blinding and to use placebo in this study.

The studied side effects of the drugs were as follows:

Desmopressin side effects: seizure (measurement of blood sodium levels, in terms of the possibility of hyponatremia), headache, nasal congestion, nausea, abdominal cramps, anuria.

Oxybutynin side effects: palpitations (reviewed electrocardiography [ECG]), drowsiness, dizziness, hives, constipation, urinary retention.

Tolterodine side effects: dizziness, weakness, abdominal pain, dysuria, urinary frequency, cough.

### Ethical issues

1) The research followed the tenets of the Declaration of Helsinki; 2) A consent form was given to all patients, and after obtaining the consent, patients were enrolled; and 3) The research was approved by the institutional review board of Mashhad University of Medical Sciences.

### Statistical analysis

After recording the results of observers and the results of study data by using descriptive statistical methods, the centrality (mean) and dispersion (standard deviation) were analyzed. To express the degree of agreement between observers, by using 2 × 2 tables and a statistical test of kappa, the kappa values ​​were calculated. To perform statistical tests (*t* and chi-square), the SPSS software was used (*P* < 0.05 was considered significant in all cases).

## Results

**Figure 1:**
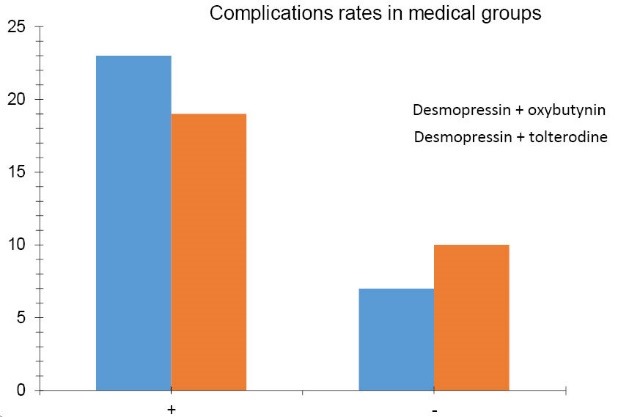
Complications rates in medical groups.

In the conducted study, among the 59 patients with primary nocturnal enuresis in age range of 5 to 14 years, who visited nephrology clinic of Dr. Sheikh pediatrics hospital of Mashhad (in 2013). The patients were divided into 2 treatment groups, the first group (n = 29) received combined therapy with desmopressin and oxybutynin, and the second group (n = 30) received combined therapy with desmopressin and tolterodine. The results showed that the mean age of patients in group of desmopressin plus oxybutynin was 2.38 ± 7.96 years and in group of desmopressin plus tolterodine was 2.71 ± 7.86 years. In group of desmopressin plus oxybutynin, 16 of 29 patients (55.1%) were male and 13 patients (44.9%) were female, and in the other group 17 of 30 patients (56.6 %) were male and 13 patients (43.4%) were female. The 2 groups did not differ in terms of sex composition (*P* = 0.908). The complication rates in medical groups are shown in Figure 1.

One-month and 3-month consequences in treatment group of desmopressin + oxybutynin:

- In the treatment group of desmopressin + oxybutynin, 21 of 29 (72.4 %) patients were recovered completely in assessing the consequences of 1-month treatment, and no improvement was observed in 8 patients (27.6%).
- In study of the consequences of 3-month treatment with desmopressin + oxybutynin, 13 patients (44.8%) achieved a complete remission, and no improvement was observed in 16 patients (55.2%).

**Figure 2:**
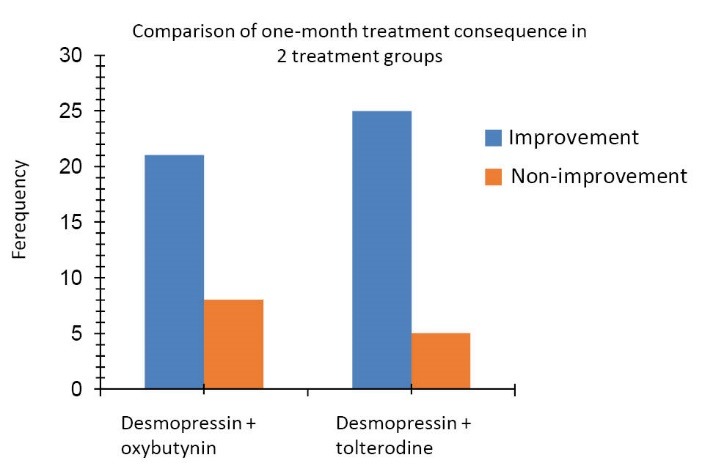
Comparison of one-month treatment consequence in 2 treatment groups.

**Figure 3:**
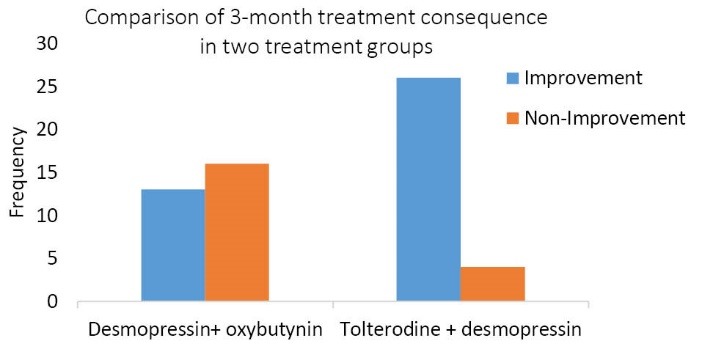
Comparison of one-month treatment consequence in 2 treatment groups.

One-month and 3-month consequences in treatment group of desmopressin + tolterodine:

- In the treatment group of desmopressin + tolterodine, in the study and evaluation of the consequences of 1-month treatment, it was found that 25 of 30 patients (83.34%) achieved complete remission, and 5 patients (16.65%) still suffered.
- In the evaluation of the consequences of 3-month treatment with desmopressin + tolterodine, it was found that 26 patients (86.7%) had complete remission, and 13.3% of individuals still suffered from enuresis.
- Comparison of the treatment group of desmopressin + oxybutynin with the treatment group of desmopressin + tolterodine in terms of 1-month and 3-month treatment outcomes:
- In the treatment group of desmopressin + oxybutynin, 21 of 29 patients were recovered completely in assessing the consequences of one month treatment (72.4 %) and in the treatment group of desmopressin + tolterodine, in the study and evaluation of the consequences of one month treatment, it was found that 25 of 30 patients (83.34%) achieved complete remission. Comparison of the 2 groups, in terms of 1-month treatment outcomes showed that there was no significant difference between the improvements of patients in the 2 groups (*P* = 0.312). Also, in study of the consequences of 3-month treatment with desmopressin + oxybutynin, 13 patients (44.8%) achieved a complete remission and no improvement was observed in 16 patients (55.2%). In the evaluation of the consequences of 3-month treatment with desmopressin + tolterodine, it was found that 26 patients (86.7%) had complete remission and 13.3% of individuals still suffered from enuresis. Considering the results, significant differences were observed between the 2 groups such that the treatment with desmopressin + tolterodine was more effective than treatment with desmopressin + oxybutynin (*P *= 0.001).
- There is no significant relationship between gender and improvement in one-month treatment, in a way that the response to treatment in total was 78%, 84.6% in girls and 72.7% in boys (*P *= 0.274). There is no significant relationship either between gender and improvement in 3-month treatment, in a way that the response to treatment in total was 66%, 73% in girls and 60% in boys (*P *= 0.315).
- There is no significant relationship between age and improvement in one-month treatment, in a way that the response to treatment in total was 78%, in age group of 7-5 years 80.6%, in the age group of 8-10 years old 72.2% and in the age group of 11-14 years old was 80% (*P *= 0.779). There is no significant relationship between age and improvement in 3-month treatment, in a way that the response to treatment in total was 66%, in age group of 7-5 years 67.7%, in age group of 10-8 years old 61.1%, and in the age group of 11-14 years old was 70% (*P *= 0.859).
- There is no significant relationship between side effects and improvement in one-month treatment, in a way that the response to treatment in total was 78%, in children who have suffered from side effects 76.6% and in children without experiencing the side effects was 81.2% (*P *= 0.710). There is no significant relationship between side effects and improvement in 3-month treatment, in a way that the response to treatment in total was 66.1%, in children who suffered from side effects 65.1%, and in children without experiencing the side effects was 68.7% (*P *= 0.793).
- Comparison of 1-month and 3-month consequence in 2 studied treatment groups is shown in Figures 2 and 3.

## Discussion

Enuresis, along allergic disorders, is one of the most common chronic disorders in children. Its prevalence in different regions and in children older than 5 years old is 5% to 20%, and it is due to developmental delay in the related somatic mechanisms (17,18). There are different therapeutic approaches for nocturnal enuresis, including behavioral modification, use of alarm and eventually pharmacotherapy, where in addition to early treatment; pharmacotherapy is also effective in encouraging children to continue behavioral therapy. Among the drugs which can be used, we can name desmopressin and tricycle antidepressant drugs. Anticholinergic drugs that reduce detrusor activity are not alone effective for the treatment of nocturnal enuresis and should be used in combination with other therapies (e.g., in combination with desmopressin) (19). It should be noted that desmopressin is available in 2 forms nasal spray and oral tablet where the oral form of the drug is recommended for short-term effects and fewer side effects (20). By reducing the production of urine at night, desmopressin can improve nocturnal enuresis, and the children who need to be treated by desmopressin or desmopressin along with an anticholinergic are poly uric. Tricyclic antidepressant drugs such as Imipramine, which is also used for the treatment, are very effective in reducing the number of wet nights. But children have often relapses after discontinuing the use of the drug (19).

In the present study, we have accommodated 59 patients with primary nocturnal enuresis into 2 treatment groups of treatment with desmopressin and oxybutynin and therapy with desmopressin and tolterodine and we examined therapeutic responses after one month and after 3 months. In the treatment group of desmopressin and oxybutynin 21 of 29 patients were recovered completely in assessing the consequences of 1-month treatment (72.4 %) and no improvement was observed in 8 patients (27.6%) In study of the consequences of 3-month treatment with this group 13 patients (44.8%) achieved a complete remission and no improvement was observed in 16 patients (55.2%). In the treatment group of desmopressin and tolterodine, in the study and evaluation of the consequences of 1-month treatment, it was found that 25 of 30 patients (83.34%) achieved complete remission and 5 patients (16.65%) were suffering from the same disease. In the evaluation of the consequences of 3-month treatment with this group, it was found that 26 patients (86.7%) had complete remission and 13.3% of individuals still suffered from enuresis.

In the statistical analysis, there was no significant difference between the groups after one month, but in 3 months of treatment, statistically, significant differences were observed between the 2 groups, such that desmopressin plus tolterodine therapy was more effective. In this study, no statistically significant relationship was observed between age and recovery, gender and improvement, number of family members and improvement in study of consequences of the 1-month and 3-month treatments.

Austin et al (21) conducted a research work in 2008 to study and compare the effects of desmopressin alone and desmopressin plus tolterodine. In that study, they examined 41 patients with resistant nocturnal enuresis to desmopressin treatment, in whom Intestinal problems and lower urinary tract dysfunction were rejected. During 4 weeks, the patients were treated in 2 groups. In one of the groups, placebo + desmopressin and in the other tolterodine + desmopressin were used. Then, the diagram of volume- frequency for 3 days and the number of wet nights were recorded for each patient in one week. In that study, the complete response was defined as the absence of even one wet night in a week and partial response was defined as a decrease of more than 90% in the number of wet nights in a week. After one month of treatment, the mean of dry nights in the group treated with desmopressin plus tolterodine was significantly higher than the mean of the group who only received desmopressin, such that the amount of bed wetting in a group that received tolterodine was 66% lower than the group who were treated with mono-therapy regimen. The results of that study were partly consistent with our results, and the difference is due to the fact that in our study, other anticholinergics (oxybutynin) were used instead of placebo. Tolterodine is used for urinary incontinence in adults and has fewer side effects than oxybutynin.

Munding et al (22) studied safety and efficacy of this drug in children. They treated 30 children aged 4 to 17 years with primary enuresis with an adult dose of tolterodine (1 mg as twice a day). Among them, 33% had complete remission and 40% had partial remissions. Thirteen percent suffered from minor complications of the drug, and only one person stopped treatment because of diarrhea. None of the children showed symptoms such as hyperpyrexia, flushing and intolerance to sunlight, which demonstrated that tolterodine can be easily applied in children to treat enuresis. Those results are somewhat consistent with our study on the effectiveness of tolterodine, and differences in methodology justify differences in the obtained results.

Rombis et al (23) studied during the 4 years 142 children with enuresis in age range of 6.5 to 18 years that included 93 boys and 49 girls. Eight of these children also had enuresis during the day. In this comprehensive study, initially the type of enuresis (primary or secondary) was determined and then the children were divided in several treatment groups. In one group, 60 children were treated only by behavioral therapy, and in other groups, 50 children with detrusor instability were treated with oxybutynin or tolterodine alone, 20 children with daily and nightly enuresis were treated with desmopressin plus oxybutynin or desmopressin plus tolterodine, and the remaining 91 children were treated with desmopressin alone. These children were assessed 3 and 6 months later. Out of 91 children who had received desmopressin as the only treatment, 66 of them were dried and 28 of them had relapsed again in 2 weeks, where in this case the treatment continued for 3 months. Among them, nine children were completely dry. In other groups, the response to complete treatment was observed. Among the reasons for the differences between results of this study with our study, we can mention the large sample size and longer follow-up. Also in this study, only children with increased activity of detrusor were treated with anticholinergic, which justifies the complete response to treatment in both treatment groups of desmopressin + oxybutynin and treatment group of desmopressin + tolterodine.

Neveus et al, in a study compared renal concentrating capacity and functional capacity of bladder among 55 dry children as control group and children with mono-symptomatic enuresis in several groups, which comprise the group that only received desmopressin, the group that only received oxybutynin, and the group that received the combined therapy of these 2, and the group which was resistant to treatment. Results of this study showed that children who responded to oxybutynin had smaller bladder or hyperactive detrusor, while those who responded to desmopressin or combined treatment induced had enuresis due to polyuria (24). Also in other study, Zaffanello et al examined the effect of tolterodine in the enuresis resistant to treatment. He treated 27 children with refractory to alarm and desmopressin in 3 groups and for 5 weeks. In the first group, he used placebo, in the second group tolterodine with dose of 1-2 mg per day, and in the last group, he used imipramine with dose of 25-50 mg at bedtime. In this study, one person was dried spontaneously in the beginning of the study and one was excluded because of side effects. Among the remaining 25 children, the number of wet nights with placebo, tolterodine and imipramine were 11 (+/- 3.9), 10.4 (+/- 3.9) and 7.8 (+/- 5.1), respectively. According to the analysis of findings, imipramine was markedly better and more effective than placebo and tolterodine, and in monotherapy, tolterodine had no proven effects. Nine children were suffering from side effects with imipramine and one child showed side effects with tolterodine, which indicates that despite the effectiveness of imipramine, emergence of side effects are common with this drug (18). The differences between the results of this study with our study can be due to the use of a smaller sample size and the use of tolterodine without desmopressin. In this regard, we can refer to the study of Martin et al (25), which was conducted to evaluate the effectiveness of the use of 2 drugs of desmopressin and oxybutynin together. The results showed significant difference between the mean of bladder compliance and pressure of urine output from the bladder in patients treated with oxybutynin with an excellent and partial response to treatment. It was reported that the therapeutic effect of 2 drugs desmopressin and oxybutynin together in these patients was much more better that the effect of oxybutynin alone.

In the study of Lee et al (26), that was conducted with the aim of evaluating combined therapy with desmopressin and oxybutynin in children with enuresis, compared with monotherapy, 158 children with enuresis were examined at 1, 3, and 6 months of treatment based on the average frequency of enuresis. Among these, 47% of children had mono-symptomatic enuresis and others had poly-symptomatic enuresis. The results showed that combined treatment had the best and fastest results, regardless of the fact that children were mono-symptomatic or poly-symptomatic and was much more cost effective than monotherapy.

In the study of Falak-ul-Aflaki et al (27), the effect of desmopressin on nocturnal enuresis in children of 5 to 14 years old were studied and ultimately complete remission was observed in 82.5% of patients. 17.5% had no recovery and no significant difference was observed between age, sex and level of stress. The effect of the drug on enuresis in children and also in this study the response rate of children with a positive family history of enuresis was more favorable towards therapeutic effects of the drug. In our study also no significant differences were observed between age and gender and the effect of desmopressin along with anticholinergics on enuresis in children.

## Conclusion

Nocturnal enuresis is one of the most common problems in children and due to its various negative effects; several treatment methods have been created. In the present study, we have examined therapeutic effect and the roles of 2 anticholinergics of oxybutynin and tolterodine as a combined treatment with desmopressin in patients with nocturnal enuresis. The results indicate that tolterodine along with desmopressin has significantly acted better in complete remission of patients in evaluation of the 3-month treatment than the treatment with desmopressin and oxybutynin. Also in terms of occurrence of complications, no difference was seen between the two groups.

## Limitations of the study

A limitation of the current study was the small sample of convenience and some of the children and parents did not cooperate with us and we could not follow up them.

## Authors’ contribution

All authors contributed to design of the research. AA, ME, MN, FG, and YR conducted the research. ABH and SES analyzed the data. MV and NG prepared the manuscript. All authors read, revised, and approved the final manuscript.

## Conflicts of interest

The authors declared no competing interests.

## Ethical considerations

Ethical issues (including plagiarism, misconduct, data fabrication, falsification, double publication or submission, redundancy) have been completely observed by the authors.

## Funding/Support

This paper has been derived from the student’s thesis of Dr Neda Ghanei. This study was granted by Mashhad University of Medical Sciences (grant No. 1302).
